# Seed phytochemicals shape the community structures of cultivable actinobacteria‐inhabiting plant interiors of Thai pigmented rice

**DOI:** 10.1002/mbo3.591

**Published:** 2018-03-25

**Authors:** Nareeluk Nakaew, Rungroch Sungthong

**Affiliations:** ^1^ Department of Microbiology and Parasitology Faculty of Medical Science Naresuan University Phitsanulok Thailand; ^2^ Infrastructure and Environment Research Division School of Engineering University of Glasgow Glasgow United Kingdom

**Keywords:** actinobacteria, bacterial community, bioactivity, endophytes, phytochemicals, pigmented rice

## Abstract

We examined abundance, bioactivity, and endophytism of cultivable actinobacteria isolated from plant interiors of two Thai pigmented rice cultivars: Hom Nin (HN) rice and Luem Pua (LP) glutinous rice. Both rice cultivars housed the same amount of endophytic actinobacteria (33 isolates each). *Microbispora* (76%) and *Streptomyces* (73%) were the predominant endophytic actinobacteria of LP glutinous rice and HN rice, respectively. *Sphaerisporangium* (9%) was found only in LP glutinous rice. Twelve percent of endophytic actinobacteria was the possibility of discovering novel species from both rice cultivars. Most endophytic actinobacteria exhibited plant growth‐promoting potentials, including antimicrobial activity against test bacteria and phytopathogenic fungi, solubilization of phosphate, and production of biostimulants (i.e., ammonia, indole‐3‐acetic acid, and siderophore) and biocatalysts (i.e., amylase, cellulase, chitinase, lipase, and protease). Our findings revealed that seed phytochemicals of pigmented rice (e.g., anthocyanin, γ‐oryzanol, phytate, antioxidants, and content of amylose) were effectors, shaping the community structures and biofunctions of endophytic actinobacteria. We conclude that pigmented rice is yet a challenging source for discovery of bioactive and novel actinobacteria. This study also provides new insights into the plant‐endophyte interactions by which seed phytochemicals act as a primary checkpoint in the natural selection for establishing unique plant endophytomes.

## 1 INTRODUCTION

Microbes inhabiting plant interiors, so‐called “endophytes,” are among the best‐known plant‐microbe interactions (Hardoim et al., 2015). Ecological lifestyles of endophytes are rather mutualism or commensalism with their host plants than parasitism (Hardoim et al., 2011; Mano & Morisaki, 2008). The endophytism is believed to take place through natural selection, depending upon the genetic variety of host plants and microbial species (Hardoim et al., 2011, 2015; Mano & Morisaki, 2008). It is conceivable that different species of host plants or even across different varieties of a single host plant species reveal dissimilar endophytic microbiomes (Hardoim et al., 2011, 2015; Mano & Morisaki, 2008; Rangjaroen, Rerkasem, Teaumroong, Sungthong, & Lumyong, 2014). Hence, the genetic diversity of host plants is a prime factor, regulating distinct plant metabolomes that is supposed to be a set of environmental cues in the screening of mutual microbes. Plant root exudation is an example mechanism of how do different plants establish their microbial association (Hardoim et al., 2011; Hartmann, Schmid, van Tuinen, & Berg, 2009). Phytochemicals consisted in root exudates act as the sorting tools, being either attractants or repellents toward the rhizosphere microbial community. However, roles of *in planta* phytochemicals on the selection, subsistence, and distribution of endophytic microbes are rarely known.

Interestingly, numerous studies report the bioactive metabolites that are produced either by endophytes or their host plants (Golinska et al., 2015; Qin, Xing, Jiang, Xu, & Li, 2011; Strobel & Daisy, 2003). The endophytes dwelling in such chemically bioactive niche within plant materials would indicate the microbial evolution through genetically resistant mechanisms, for example, sharing biosynthetic genes for bioactive metabolites or harboring cellular mechanisms and genes to resist foreign bioactive substances. There are many studies that have disclosed the bioactive functions of endophytes that act as the secondary defensive tools in plant immunology for optimizing plant growth and protecting plants from phytopathogens and harmful xenobiotic agents like herbicides (Golinska et al., 2015; Kampapongsa & Kaewkla, 2016; Qin et al., 2011; Rangjaroen et al., 2017; Strobel & Daisy, 2003; Tétard‐Jones & Edwards, 2016; Tian et al., 2004). Out of such plant growth‐promoting benefits, various metabolites from endophytes also reveal pharmaceutical and medical potentials, for example, antimicrobial, antiviral, and anticancer/antitumor activities (Golinska et al., 2015; Qin et al., 2011; Strobel & Daisy, 2003). Hitherto, endophytes are yet a promising source for discovering novel and effective medicines. Although diverse endophytes can synthesize bioactive compounds, we still keep focusing on actinobacteria (Golinska et al., 2015; Kampapongsa & Kaewkla, 2016; Qin et al., 2011; Tian et al., 2004) that are notable producers of various commercial metabolites and promising reservoirs for novel drug discovery.

For centuries, rice (*Oryza* spp.) is an important crop cultivated worldwide for human nourishment (Hardoim et al., 2011; Mano & Morisaki, 2008; Rangjaroen et al., 2017; Tian et al., 2004). The trend of pigmented rice consumption has been increasing because of its rich nutritive values, especially for antioxidants and vitamins, when compared to the other white rice (Goufo & Trindade, 2014; Sompong, Siebenhandl‐Ehn, Linsberger‐Martin, & Berghofer, 2011). In this study, we aim to isolate cultivable actinobacteria that live in plant interiors of different pigmented rice cultivars and addressing the impacts of phytochemical properties of the housing plant tissues on the community structures of such endophytic actinobacteria. Two commercial Thai pigmented rice cultivars: Hom Nin (HN) rice and Luem Pua (LP) glutinous rice serve as the host plants for studying their cultivable‐based endophytic actinobacteria. The isolated endophytic actinobacteria were subjected to the bioactivity screening for their modes of action in promoting plant growth. Lastly, we characterized some phytochemical properties of rice plant tissues and linked the possible impacts of such phytochemicals on the community structures and biofunctions of rice endophytic actinobacteria.

## 2 RESULTS AND DISCUSSION

### 2.1 Endophytic actinobacteria of Thai pigmented rice and their bioactivities

**Figure 1:**
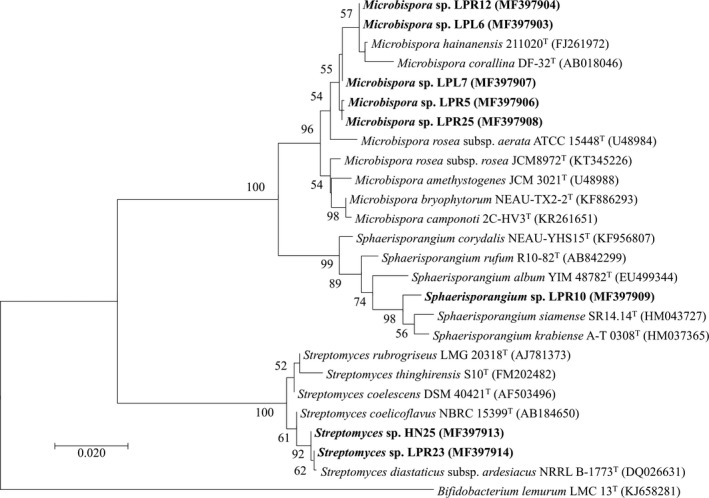
Maximum likelihood tree constructed with nearly full‐length 16S rRNA gene sequences of the representative rice endophytic actinobacteria (see Tables 1 and 2 for more information) and their closely related phylogenetic species. HN and LP refer to Hom Nin rice and Leum Pua glutinous rice, respectively. *Bifidobacterium lemurum* was the out‐group for this phylogenetic analysis. Bootstrap values (>50%) based on 1000 replications are shown at branch nodes, and scale bar represents 0.02 substitutions per nucleotide position. The code in parenthesis refers to the accession number in GenBank database

**Figure 2:**
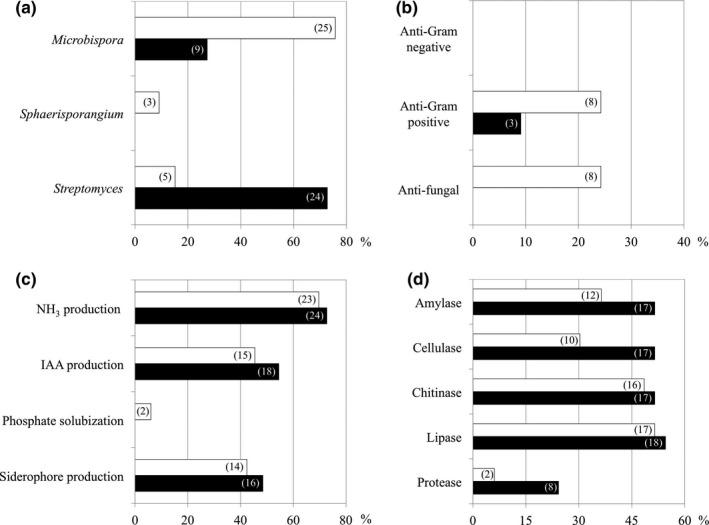
Generic diversity of endophytic actinobacteria and their plant growth‐promoting potentials. The generic abundance (a) was determined using morphological and phylogenetic characteristics of endophytic actinobacteria (see Table 2). The plant growth‐promoting potentials comprised of antimicrobial activity (b), soil nutrient and mineral conversion and biosynthesis of plant biostimulants (c), and production of some biocatalysts (d). White and black bars are results derived from Leum Pua glutinous rice and Hom Nin rice, respectively. The number in parenthesis refers to the isolate number of endophytic actinobacteria

An equal number (33 isolates) of endophytic actinobacteria was isolated from each Thai pigmented rice cultivar (Table 1). All obtained endophytic actinobacteria were only derived from rice seedlings grown in agricultural soil. Based on morphological characteristics, all endophytic actinobacteria were categorized into three groups and identified with the phylogenetic analysis of their 16S rRNA gene sequences into three genera; *Microbispora*,* Sphaerisporangium*, and *Streptomyces* (Table 2, Figures 1 and 2a). *Microbispora* (76%) and *Streptomyces* (73%) was the predominant endophytic actinobacterium of LP glutinous rice and HN rice, respectively. *Sphaerisporangium* (9%) was found only in LP glutinous rice. It is conceivable that *Microbispora* and *Streptomyces* are typical endophytic actinobacteria of rice plants (Kampapongsa & Kaewkla, 2016; Tian et al., 2004). The chance to discover novel species of endophytic actinobacteria from any pigmented rice cultivar was estimated at 12.5%. With such level, we still believe that pigmented rice would be a promising source offering unique ecological niches for exploring novel actinobacteria.

**Table 1:** Endophytic actinobacteria isolated from Thai pigmented rice

Rice cultivar	Plant material^a^	Code of endophytic actinobacteria	No. of isolate
Hom Nin (HN) rice			
	Root	HN1‐4, 14‐33	24
	Stem	HN5‐12	8
	Leaf	HN13	1
			ΣIsolates = 33
Leum Pua (LP) glutinous rice			
	Root	LPR1‐26	26
	Stem	–	0
	Leaf	LPL1‐7	7
			ΣIsolates = 33

a The 15‐day‐old rice seedlings derived from soil cultivation are the sources of these plant materials.

**Table 2:** Morphological and phylogenetic characterization of endophytic actinobacteria isolated from Thai pigmented rice

Morphological characteristics^a^					Phylogenetic characteristics^b^		
Morphological group(No. of isolate)	Colony color	Spore color	Spore number	Soluble pigment	Group representative (GenBank accession no.)	% Identity^c^	Closest phylogenetic species (GenBank accession no.)
Group I (34): HN7, 10, 12, 16–20, 27 (9) LPR1–3, 5, 7, 9, 11–19, 22, 24, 25, LPL1–7 (25)	Reddish brown	White	Pair of spores	Not produced	HN10 (MG687439) LPL6 (MF397903) LPL7 (MF397907) LPR5 (MF397906) LPR12 (MF397904) LPR25 (MF397908)	99.17 99.72 99.31 99.17 99.79 99.24	*Microbispora hainanensis* 211020^T^ (FJ261972)
Group II (3): LPR4, 8, 10	Red	White	Spherical spore	Produced in red	LPR4 (MG687442) LPR10 (MF397909)	98.72 98.65	*Sphaerisporangium siamense* SR14.14^T^ (HM043727)
Group III (29): HN1–6, 8, 9, 11, 13–15, 21–26, 28–33 (24) LPR6, 20, 21, 23, 26 (5)	Cream	Gray	Chain of >20 spores	Not produced or Produced in red	HN2 (MG687436) HN3 (MG687437) HN6 (MG687438) HN22 (MG687440) HN25 (MF397913) HN33 (MG687441) LPR23 (MF397914) LPR26 (MG687443)	99.71 99.65 99.72 99.65 99.65 99.65 99.72 99.65	*Streptomyces diastaticus* subsp. *ardesiacus* NRRL B‐1773^T^ (DQ026631)

a Morphological characterization was carried out after growing endophytic actinobacteria on HT agar medium for 14 days.

b The 16S rRNA gene sequences of the group representatives were deposited in GenBank and used for phylogenetic analysis (Figure 1).

c The % identity refers to the percentage of the gene sequence similarity of the group representatives compared to their closest phylogenetic species.

Every endophytic actinobacterium obtained was evaluated for their bioactivities in promoting plant growth (Figure 2b–d). None of the endophytic actinobacteria inhibited the growth of gram‐negative bacteria tested (Figure 2b). Twenty‐four percent of endophytic actinobacteria derived from LP glutinous rice showed anti‐gram‐positive and antifungal activities, which was higher than those (0%–9%) of HN rice. While, slightly higher number (48%–73%) of endophytic actinobacteria isolated from HN rice could produce ammonia, IAA, and siderophore, compared to those (42%–70%) of LP glutinous rice (Figure 2c). Only a few percentage (6%) of endophytic actinobacteria could solubilize phosphate, and all of them derived from LP glutinous rice. Interestingly, many isolates of endophytic actinobacteria were capable of producing biocatalysts (Figure 2d). The higher number (24–55%) of endophytic actinobacteria derived from HN rice could produce biocatalysts, compared to those (6–52%) of LP glutinous rice. These findings did not surprise us, as most endophytic actinobacteria are the best‐known sources of bioactive metabolites (Golinska et al., 2015; Kampapongsa & Kaewkla, 2016; Qin et al., 2011; Tian et al., 2004). However, further studies for the structural elucidation and additional bioactivity screening of bioactive metabolites derived from such emerging endophytic actinobacteria would enhance the possibility of discovering novel metabolites for pharmaceutical and agricultural applications.

### 2.2 Endophytic colonization and role of phytochemicals in endophytism

The highest number (24–26 isolates) of endophytic actinobacteria inhabited the seedlings’ root tissues of both rice cultivars (Table 1). Although we found abundant endophytic actinobacteria in rice roots, another study reports more found in rice leaf compartments (Kampapongsa & Kaewkla, 2016). It might be an influence of the difference in growth and developmental phases of rice, as we used 15‐day‐old seedlings, while another (Kampapongsa & Kaewkla, 2016) used mature rice plants (7‐week‐old planting in paddy fields). In fact, plant growth during the seedling stage is highly dependent upon the root functions rather than the photosynthesis. Hence, roots of rice seedlings that have direct contact with soil, and would be rich in nutrients and minerals, were optimal and attractive for the endophytic colonization of actinobacteria (Hardoim et al., 2011; Hartmann et al., 2009). Some studies reveal that rice seeds house various bacterial endophytes, where they prolong their life in plant interiors during growth and development of rice and pass through the next generation of rice seeds (Hardoim, Hardoim, van Overbeek, & van Elsas, 2012; Kaga et al., 2009). In this study, there was no endophytic actinobacterium isolated from peeled‐off mature seeds of both pigmented rice cultivars. It might be because of the influences caused by the differences in rice plant species and phytochemical properties of seeds used for the studies. For the distribution patterns and rates within the upper‐ground interiors of both rice cultivars, endophytic actinobacteria were found more abundant in stems for HN rice but in leaves for LP glutinous rice (Table 1). With the same age and length of rice seedlings, *Microbispora* showed the fastest distribution to the leaf interiors of LP glutinous rice (seven isolates, Tables 1 and 2). The distribution rates seemed slower in HN rice because most of the endophytic actinobacteria stayed in stem interiors (*Microbispora* 3 isolates and *Streptomyces* 5 isolates (Tables 1 and 2)). Such differences might be a result of the variation in morphological characteristics of endophytic actinobacteria. *Microbispora* (1.0–1.4 to 1.2–1.7 μm in Ø of a pair of spores) and *Streptomyces* (0.5–2.0 μm in Ø of vegetative hyphae) have relatively smaller in their morphology compared to *Sphaerisporangium* (Goodfellow et al., 2012). Whilst, *Sphaerisporangium* produces a large spore vesicle (~1.5–8.0 μm) (Goodfellow et al., 2012), a possible reason as to why we found this genus only in roots. Another study also proposed a novel species of this genus; *Sphaerisporangium rufum* isolated from root tissues of rice (Mingma et al., 2014).

It was affirmable that all actinobacteria obtained were indeed endophytes of both rice cultivars and would have their origin solely from soil and water used for cultivation of rice seedlings, as we used the surface sterilized mature seeds (without peeling) for production of such rice seedlings. Besides, no actinobacterium was isolated from any plant tissues derived from the cultivation in an axenic moist chamber. The absence of actinobacterium in the last washes (controls) derived from the surface sterilization of any rice plant tissues also supported the rice interior‐borne actinobacteria, which was consistent with the PCR confirmation of such last washes (data not shown). By the way, the abundances of *Microbispora* and *Streptomyces* in both rice cultivars were apparently different (Figure 2a). These suggested that the mechanisms by which rice plants select neighbor actinobacteria for establishing endophytism were highly concerned with the difference in phytochemical properties of rice tissues. The phytochemicals of polished rice seeds of both pigmented rice cultivars are listed in Table 3, while the seedling phytochemicals of both rice cultivars were analyzed and reported in this study (Table S1). The phytochemical profiles of rice seedlings were similar between both rice cultivars, while some of their seed phytochemicals were significantly different. We discovered that the difference in seed phytochemicals would be a pioneer set of effectors, inducing neighbor microbes to set up their life within plant interiors. Although seeds of pigmented rice contained rich contents of bioactive phytochemicals, for example, anthocyanin, γ‐oryzanol, phytate, and antioxidants (Goufo & Trindade, 2014; Sompong et al., 2011), we found that such phytochemical contents were highest in seeds of LP glutinous rice. We also observed some links between the richness of such bioactive phytochemicals and biofunctions of endophytic actinobacteria. For example, more isolates of endophytic actinobacteria derived from LP glutinous rice showed antimicrobial activities than those of HN rice, supporting the hypothesis that chemically active plant materials would offer a unique ecological niche for the discovery of bioactive endophytes. Another example was the richness of phytate (4800 mg·kg^−1^) in LP glutinous rice seeds. Phytate is a phosphate (P)‐storage form of plants, and that is the reason why we found P–solubilizing endophytic actinobacteria only in this rice cultivar. Moreover, the low or negligible content of amylose is the best‐known characteristic of glutinous rice (Olsen & Purugganan, 2002; Sompong et al., 2011). With this notable, we, therefore, found fewer amylase‐producing endophytic actinobacteria from LP glutinous rice than those of HN rice (Figure 2d). Based on our findings, rice plants employed variable phytochemicals during seed germination as the natural selection tools for attracting and shaping the community structures of their pioneer endophytic actinobacteria.

**Table 3:** Some phytochemicals of polished rice seeds^a^

Phytochemical^b^	Unit	Hom Nin rice	Leum Pua glutinous rice
Fat			
Saturated fat	g·100·g^−1^	0.92	0.76
Unsaturated fat	g·100·g^−1^	1.79	2.35
Polyunsaturated fat (ω−3, ω−6)	g·100·g^−1^	0.88	1.19
Monounsaturated fat (ω−7, ω−9)	g·100·g^−1^	0.91	1.16
Carbohydrate			
Dietary fiber	g·100·g^−1^	6.89	2.33
Protein			
Dry basis	%	10.53	10.63
Wet basis	%	9.43	9.46
Collagen	mg·kg^−1^	<50	<50
Vitamin B_1_	mg·100·g^−1^	0.33	0.05
Vitamin B_2_	mg·100·g^−1^	0.028	0.035
Vitamin B_3_	mg·100·g^−1^	5.78	6.48
Vitamin B_9_	μg·g^−1^	<0.78	<0.78
Vitamin B_12_	μg·100·g^−1^	<0.10	<0.10
Vitamin E			
α‐Tocopherol	mg·kg^−1^	7.78	16.83
γ‐Tocopherol	mg·kg^−1^	11.3	6.48
δ‐Tocopherol	mg·kg^−1^	0.75	0.39
Anthocyanin	mg·100·g^−1^	1.44	46.56
γ‐Oryzanol	mg·kg^−1^	411.90	490.49
Phytate	mg·kg^−1^	2861.13	4801.15
Total antioxidant	mg ascorbic acid 100·g^−1^	192.57	833.77
Calcium (Ca)	mg·kg^−1^	121.90	169.75
Iron (Fe)	mg·kg^−1^	13.30	84.18
Manganese (Mn)	mg·kg^−1^	22.25	35.38
Zinc (Zn)	mg·kg^−1^	23.75	23.60

a The polished rice seeds used for phytochemical analysis were younger than 4 months old after harvest.

b The phytochemical properties were analyzed by and obtained from the Bureau of Rice Research and Development, Thailand (available for searching at http://www.brrd.in.th/library/images/stories/pdf/brrd5501001c2.pdf).

We conclude that pigmented rice is yet a promising source for discovery of bioactive and novel actinobacteria, which are reservoirs of diverse biotechnological metabolites. This study also provides new insights into the plant‐endophyte interactions by which plant seed phytochemicals would act as a primary checkpoint in the natural selection process for establishing plant endophytomes.

## 3 EXPERIMENTAL PROCEDURES

### 3.1 Source and preparation of plant materials

HN rice and LP glutinous rice are local rice breeds cultivated routinely for commercial purpose at Khao Kho district, Phetchabun province, Thailand. The mature seeds of both rice breeds were purchased from their cultivating area mentioned above and maintained in the dry condition prior use (within 3 months after harvest). Plant materials used for isolation of endophytic actinobacteria comprised of 20 peeled‐off mature seeds, six seedlings grown in an axenic moist chamber for ~10 days (divided into roots and shoots), and six seedlings planted in an agricultural soil for ~15 days (divided into roots, stems, and leaves). The growing conditions of rice seedlings (i.e., dark‐light cycle, moisture, soil, and watering) were controlled the same for both rice breeds. The rice seedlings derived from soil cultivation were gently uprooted, and their roots were cleaned several times under running tap water. After thoroughly washing plant materials with tap water, their surfaces were sterilized by soaking in 10% (w/v) sodium hypochlorite solution for 5 min and washing twice with sterile distilled water.

### 3.2 Isolation of endophytic actinobacteria from plant materials

Surface sterilized plant materials were ground separately in the presence of 1 ml sterile distilled water, using aseptic mortar and pestle. Starch Casein agar (Table S2) plus nalidixic acid (25 μg·ml^−1^) and cycloheximide (10 μg·ml^−1^) was an isolation medium for this study. The medium was spread over with a 100 μl of the ground plant suspension prepared above. Six seeded agar plates per each plant material were carried out and incubated at 30°C for 20 days, while the other agar plates seeded with the last washes of plant materials served as controls. Appeared colonies of actinobacteria were collected and subcultured on Hickey–Tresner (HT) agar (Table S2) until becoming pure cultures. The purified isolates of actinobacteria were conserved in 20% (v/v) glycerol for a long‐term storage and further studies.

### 3.3 Morphological and phylogenetic characterization of endophytic actinobacteria

All endophytic actinobacteria obtained were grouped based on some of their morphological characteristics (Table 1), following the standard of Bergey's Manual of Systematic Bacteriology (Goodfellow et al., 2012). The representatives of each morphological group were identified at the generic level, using their 16S rRNA gene sequence data. Initially, we increased the biomass of endophytic actinobacteria by growing them in 50 ml International *Streptomyces* Project medium II (ISP2) (Table S2) under shaking incubation at 150 rpm, 30°C for 7 days. The biomass was collected by centrifugation at 12,000*g* for 5 min and used as a DNA template of 16S rRNA gene in polymerase chain reaction (PCR).

We used Phire Plant Direct PCR Kit (Thermo Fisher Scientific Inc., USA) for PCR, following the manufacturer protocol. PCR reagents (20 μl) comprised of 10 μl 2 ×  Phire Plant PCR Buffer, 0.4 μl Phire Hot Start II DNA Polymerase, 0.5 μl DNA template, 0.5 μl each universal primer (27F: 5′ AGAGTTTGATCMTGGCTCAG 3′ and 1492R: 5′ TACGGYTACCTTGTTACGACTT 3′), and 8.1 μl nuclease‐free water. PCR was carried out using a thermocycler with the following condition: 5 min predenaturation at 98°C, 40 cycles of 5 s denaturation at 98°C, 1 min annealing at 55°C, and 20 s extension at 72°C, and 1 min final extension at 72°C.

PCR products were further sequenced to retrieve their nucleotide sequencing data with a commercial service provided by Macrogen Inc., Republic of Korea. The gene sequences obtained were checked using BioEdit (http://www.mbio.ncsu.edu/BioEdit/bioedit.html) and compared to the public databases available in BLASTn (blast.ncbi.nlm.nih.gov/Blast.cgi) and EZBioCloud (http://www.ezbiocloud.net). The highly related gene sequences were collected and aligned with MUSCLE and consequently used for constructing the phylogenetic tree by MEGA7 (http://www.megasoftware.net).

A trend for discovering novel species of endophytic actinobacteria from any pigmented rice in this study was estimated. It is conceivable that many member species of several bacterial genera reveal relatively high sequence similarity of 16S rRNA gene up to 100%, but showing lower than 70% DNA–DNA relatedness values. In 2006, taxonomists suggested the species demarcation of prokaryotes at 98.2%–99.0% similarity threshold of the 16S rRNA gene sequence, which was consistent with a further in silico study at 98.65% (Kim, Oh, Park, & Chun, 2014). We, therefore, estimated here the percentage of novel actinobacteria that exhibited lower than 99% similarity of their 16S rRNA gene sequences compared to their closest related phylogenetic species.

### 3.4 Bioactivity screening of endophytic actinobacteria

Plant growth‐promoting potentials of all endophytic actinobacteria were determined with their bioactivities, comprised of antimicrobial activity, soil nutrient and mineral conversion, and production of some biostimulants and biocatalysts. The antimicrobial activity against a set of test microorganisms (i.e., bacteria and phytopathogenic fungi listed in Table S3) was evaluated using agar well diffusion screening assays. Firstly, every actinobacterial isolate was grown in HT agar medium at 30°C for 10 days. The seeded HT agar was punched with a sterile Pasteur pipette (6 mm Ø) to prepare the actinobacterial inoculum. Three actinobacterial colony‐containing agar plugs were grown further in 100 ml ISP2 broth under shaking condition at 150 rpm, 30°C for 15 days. The cell‐free culture (CFC) broth prepared by centrifugation of the actinobacterial culture in ISP2 broth at 12,000*g* for 15 min was used for antimicrobial screening against the test microorganisms.

The test bacteria were initially grown in Nutrient broth (Himedia, India) at 37°C for 5–6 hr and their cell densities were adjusted to 0.5 McFarland turbidity standard (corresponding to 10^8^ colony‐forming units (CFU) ml^−1^) by dilution with 0.85% (w/v) saline. The bacterial suspension prepared was spread over Mueller Hinton agar medium (Himedia, India). For the test fungi, the mycelium of each fungal strain was inoculated at the center of Potato Dextrose agar medium (Himedia, India). The agar plates seeded with the test microorganisms were punched to form wells with the sterile Pasteur pipette. Each agar well was loaded with 50 μL of either the CFC broth of each actinobacterial isolate, positive controls (1 mg·ml^−1^ streptomycin for the test bacteria and 1 mg·ml^−1^ cycloheximide for the test fungi) and a negative control (ISP2 broth). The incubation conditions for the assayed agar plates were at 37°C for 24 hr (for the test bacteria) or at 30°C for 3 days (for the test fungi). The positive antimicrobial activity was determined by the inhibition (clear) zone that appeared on the test agar plates at the end of incubation.

The other plant growth‐promoting activities, including the production of ammonia (NH_3_) through peptone degradation, indole‐3‐acetic acid (IAA), and siderophore, and the solubilization of phosphate, were evaluated by screening protocols described elsewhere (Nakaew, Rangjaroen, & Sungthong, 2015). We also estimated the capability to produce some biocatalysts, that is, amylase, cellulase, chitinase, lipase, and protease, using a set of specific agar medium screening assays. Briefly, Nutrient agar medium (Himedia, India) supplemented with 2 g·L^−1^ soluble starch or 1% (v/v) tributyrin (Glogauer et al., 2011) was used for testing the amylase and lipase production, respectively. The agar medium was inoculated with the actinobacteria and incubated at 30°C for 7 days. For detecting amylase production, staining the seeded agar plates with 1% (w/v) Lugol's iodine reagent for 20 min was needed. The clear zone that appeared around the actinobacterial colony determined the positive amylase and lipase production. For the production of cellulase, chitinase, and protease, the screening assays were carried out following the protocols described by Nakaew et al. (2015).

### 3.5 Phytochemical analysis of plant materials

The phytochemical profiles of plants materials were either taken from the Bureau of Rice Research and Development, Thailand (available on 1st May 2017) or analyzed by an external service provided by the Central Laboratory (Thailand) Co., Ltd. (Chiang Mai branch). The analytical methods used by the Central Laboratory are listed in Table S1.

## CONFLICT OF INTEREST

None declared.
