# Supplementary material for: Seed phytochemicals shape the community structures of cultivable actinobacteria‐inhabiting plant interiors of Thai pigmented rice
Source: Microbiologyopen. 2018 Mar 25;7(4):e00591. doi: 10.1002/mbo3.591 (PMC6079165; doi:10.1002/mbo3.591)
Supplement: Supplementary file 1 [file MBO3-7-e00591-s001.doc]

**Supporting Information**

**Seed phytochemicals shape the community structures of cultivable actinobacteria–inhabiting plant interiors of Thai pigmented rice**

**Nareeluk Nakaew^1^ and Rungroch Sungthong^2^***

^1^Department of Microbiology and Parasitology, Faculty of Medical Science, Naresuan University, Phitsanulok 65000, Thailand

^2^Infrastructure and Environment Research Division, School of Engineering, University of Glasgow, Glasgow G12 8LT, United Kingdom

***Corresponding author:**

Rungroch Sungthong

E-mail: rungroch.sungthong@glasgow.ac.uk

Tel: +44 (0) 141 330 6311

**Table S1. Some phytochemical properties of rice seedlings.*^a^***

| **Phytochemical property***^b^* | **Analytical method***^c^* | **Unit** | **Hom Nin rice** | **Leum Pua glutinous rice** |
| --- | --- | --- | --- | --- |
| Fat | AOAC (2016) 948.15 | g 100 g^-1^ | 0.32 | 0.34 |
| Saturated fat | In house method TE-CH-208 based on AOAC (2012) 996.06 | g 100 g^-1^ | 0.13 | 0.11 |
| Unsaturated fat |  | g 100 g^-1^ | 0.19 | 0.23 |
| Polyunsaturated fat (ω–3, ω–6) |  | g 100 g^-1^ | 0.17 | 0.21 |
| Monounsaturated fat (ω–7, ω–9) |  | g 100 g^-1^ | 0.02 | 0.01 |
| Carbohydrate | Compendium of Method for Food Analysis. Thailand. 1^st^ Edition 2003 | g 100 g^-1^ | 6.96 | 6.80 |
| Protein | AOAC (2016) 991.20 | g 100 g^-1^ | 1.86 | 1.69 |
| NaCl | In house method based on AOAC (2000) 937.09 | g 100 g^-1^ | 0.28 | 0.26 |
| Calcium (Ca) | In house method TE-CH-170 based on AOAC (2016) 984.27 and 999.10 with ICP-OES | mg kg^-1^ | 631.00 | 518.00 |
| Iron (Fe) |  | mg kg^-1^ | 20.56 | 32.06 |
| Manganese (Mn) |  | mg kg^-1^ | 3.85 | 4.46 |
| Zinc (Zn) |  | mg kg^-1^ | 8.82 | 7.80 |
| Total antioxidant | DPPH method | mg eq. ascorbic acid 100 g^-1^ | 14.30 | 11.03 |
| pH | In house method based on AOAC (2010) 943.02 |  | 6.51 | 6.61 |

*^a^*Rice seedlings were obtained using soil cultivation described in text. *^b^*Every measurement was carried out with the external service provided by Central Laboratory (Chiang Mai Branch, Thailand) Co., Ltd. *^c^*The Central Laboratory performed all measurements using a set of standard methods described by the Association of Official Agricultural Chemists (AOAC). ICP-OES and DPPH refer to Inductively Coupled Plasma Optical Emission Spectrometer and Diphenylpicrylhydrazyl (Molyneux, 2004), respectively.

**Additional reference**

Molyneux, P. (2004) The use of the stable free radical diphenylpicrylhydrazyl (DPPH) for estimating antioxidant activity. *Songklanakarin J Sci Technol* **26**: 211–219.

| **Starch casein agar (SCA) medium** | **Composition in 1 L** |
| --- | --- |
| Soluble starch | 10 g |
| KH_2_PO_4_ | 2 g |
| KNO_3_ | 2 g |
| NaCl | 2 g |
| Casein | 0.3 g |
| MgSO_4_·7H_2_O | 0.05 g |
| CaCO_3_ | 0.02 g |
| FeSO_4_·7H_2_O | 0.01 g |
| Agar powder | 15 g |
| pH | 7.2 ± 0.2 |
| **International *Streptomyces* Project 2 (ISP2) medium** | **Composition in 1 L** |
| Malt extract | 10 g |
| Yeast extract | 4 g |
| Dextrose | 4 g |
| Agar powder | 20 g |
| pH | 7.2 ± 0.2 |
| **Hickey–Tresner (HT) agar medium** | **Composition in 1 L** |
| Soluble starch | 2 g |
| Peptone | 0.4 g |
| Beef extract | 0.2 g |
| Yeast extract | 0.2 g |
| Agar powder | 15 g |
| pH | 7.2 ± 0.2 |

**Table S2.** Media compositions used in this study.*^a^*

*^a^*All media were prepared and steam sterilized with an autoclave at 121 °C for 15 min.

**Table S3.** List of test organisms for antimicrobial screening.

| **Test bacteria** | | **Test fungi*^a^*** |
| --- | --- | --- |
| Gram-negative bacteria | | *Pyricularia* sp. F2 (MF946553) |
|  | *Escherichia coli* ATCC 25922 | *Exserohilum* sp. F3 (MF946554) |
|  | *Pseudomonas aeruginosa* ATCC 27853 | *Arthrinium* sp. F4 (MF946555) |
| Gram-positive bacteria | | *Colletotrichum* sp. F5 (MF946556) |
|  | *Bacillus subtilis* DMST 5871 | *Rhizopus* sp. F6 (MF946557) |
|  | *Staphylococcus aureus* ATCC 25923 |  |

*^a^*The code in parenthesis refers to the accession number in GenBank database as for retrieving the source of isolation and its partial identification using internal transcribed spacer gene sequence.
